# Macrolide-Resistant *Bordetella pertussis* Infection in Newborn Girl, France

**DOI:** 10.3201/eid1806.120091

**Published:** 2012-06

**Authors:** Sophie Guillot, Ghislaine Descours, Yves Gillet, Jérome Etienne, Daniel Floret, Nicole Guiso

**Affiliations:** Institut Pasteur, Paris, France (S. Guillot, N. Guiso);; Hospices Civils de Lyon, Bron, France (G. Descours, J. Etienne, Y. Gillet, D. Floret);; University Claude Bernard, Lyon, France (G. Descours, J. Etienne, D. Floret)

**Keywords:** Bordetella pertussis, bacteria, whooping cough, antibacterial drugs, antimicrobial resistance, macrolide resistance, respiratory infections, newborn, infants, children, adolescents, France

## Abstract

A macrolide antimicrobial drug was administered to a newborn with cough. On day 23 of hospitalization, macrolide-resistant *Bordetella pertussis* was isolated from nasopharyngeal aspirates. DNA sequencing and PCR–restriction fragment length polymorphism showed a 2047 A-to-G mutation in the 3 copies of the 23S rRNA gene. Monitoring for macrolide resistance is essential in infants <6 months of age.

*Bordetella pertussis,* the causative agent of whooping cough, continues to circulate among children and adolescents even in regions with high vaccine coverage. Antimicrobial drug treatment contributes substantially to controlling transmission of the disease. In France, the treatment of choice is clarithromycin or azithromycin, which eliminate the bacterium from the respiratory tract of the infected patient and their close contacts (*1*). To date, erythromycin resistance in *B. pertussis* has been described only in the United States (*2**–**4*). The erythromycin-resistant *B. pertussis* isolates in the United States carry an A-to-G transition at nucleotide position 2047 of the 23S rRNA gene, in a region critical for erythromycin binding.

## The Study

We report the case of an 18-day-old girl, born without complication after 39 weeks of pregnancy, weighing 3,510 g. She was brought to the emergency department of Hopital Femme Mère Enfant (Lyon, France) with a 2-day history of cough without fever and worsening status. She had not previously been ill. The mother reported a cough that had persisted for 2 weeks. Physical examination found no fever, a heart rate of 169 beats/min, a respiratory rate of 60 breaths/min, signs of retraction, and an oxygen saturation of 92% in room air. Lung examination found rales in both basal fields, with a loose cough. The leukocyte count was 26.8 cells/mm^3^, with 51% lymphocytes. Venous blood gas measurement showed a pH of 7.30, a carbon dioxide partial pressure of 7.19 kPa, and a partial oxygen pressure of 4.14 kPa. Chest radiograph showed thoracic distension without signs of condensation. Nasopharyngeal aspirates were positive for *B. pertussis* by culture and specific PCR (Cepheid, Maurens-Scopont, France).

The patient was admitted to the intermediate care unit and was given azithromycin for 3 days, but her condition worsened (respiratory rate of 90–100 breaths/min, transient episodes of bradycardia at 75–100 beats/min). She was referred to the pediatric intensive care unit (ICU) 4 days after admission. On day 5, the patient underwent a 300-mL volume exchange transfusion because of increasing oxygen requirement, despite continuous positive airway pressure (fraction of inspired oxygen 50%) and increased leukocyte count (64 cells/mm^3^). The postexchange leukocyte count fell to 20 cells/mm^3^, but the respiratory benefit was only moderate. On day 14, the leukocyte count had increased to 54 cells/mm^3^, and the oxygen requirement to fraction of inspired oxygen 40%; a second 300-mL volume exchange transfusion was administered. A second sample of nasopharyngeal aspirates was obtained and was positive for *B. pertussis* by culture and specific PCR. Clarithromycin treatment was started on day 18 and continued for 7 days.

The patient started to improve after 23 days and was transferred from the ICU to the pulmonary unit on day 35. A routine nasopharyngeal aspirate was taken, and an erythromycin-resistant strain of *Staphylococcus aureus* was cultured from this aspirate (10^5^ CFU/mL); cotrimoxazole was then administrated for 8 days. The patient recovered normal pulmonary function and was discharged on day 44. Cultures of the sample taken at discharge showed a single colony of *B. pertussis*, and clarithromycin was administered for 5 days. The patient recovered completely, and results of *B. pertussis*–specific culture and PCR with nasopharyngeal aspirates were negative for *B. pertussis* on day 98.

Three isolates were collected, one at admission (FR4929), one 18 days after azithromycin treatment (FR4930) and a third 19 days after clarithromycin treatment (FR4991). FR4929 and FR4930 were sensitive to all macrolides tested (erythromycin, clarithromycin, azithromycin), but FR4991 was resistant. The Etest MIC was >256 µg/mL for all macrolides. To our knowledge, macrolide resistance has not been found in other *B. pertussis* isolates collected in France since the late 1940s.

The 3 isolates and the erythromycin-resistant isolate A228 (*2*) from the United States were analyzed for biochemical characters and by pulsed-field gel electrophoresis, genotyping and expression of virulence as described in Bouchez et al. (*5*). The 3 isolates from France belong to the same group (group IV) as that of all *B. pertussis* isolates circulating in France since 1998 and A228. They all harbor a *PtxP3* allele, a *PtxA* allele, and a *prn* 2 allele, and expressed all tested virulence factors: pertussis toxin, adenylate cyclase–hemolysin, filamentous hemagglutinin, pertactin, and fimbrial protein type 3.

PCR amplification and DNA sequencing were used to determine whether the resistance to erythromycin was associated with a mutation in the 23S rRNA gene as described by Bartkus et al. (*2*). The sequences of ≈400 nt of the 521-bp PCR fragment were determined for the 3 isolates obtained from this patient, A228 (*2*), and the reference strain Tohama (*6*). An A-to-G mutation was found at position 2047 in the 23S rRNA gene in the genomes of FR4991 and A228 but not in the genomes of FR4929 and FR4930.

*B. pertussis* carries 3 copies of the 23S rRNA gene. To test whether all 3 copies were mutated, DNA amplified by PCR (which amplified the sequences of all 3 copies of the 23srRNA) from each isolate was digested with *BbsI*: the A2047G transition in the rRNA gene is predicted to create a *BbsI* restriction site (Figure). *BbsI* cleaved the entire DNA amplified from the erythromycin-resistant isolate FR4991, confirming the presence of a G at position 2047 in the 3 copies of the 23S rRNA gene. *BbsI* did not cleave the DNA amplified from the erythromycin-sensitive isolates FR4929 and FR4930 indicating that they have no A2047G mutation in any of the 3 copies of the 23S rRNA gene.

**Figure:**
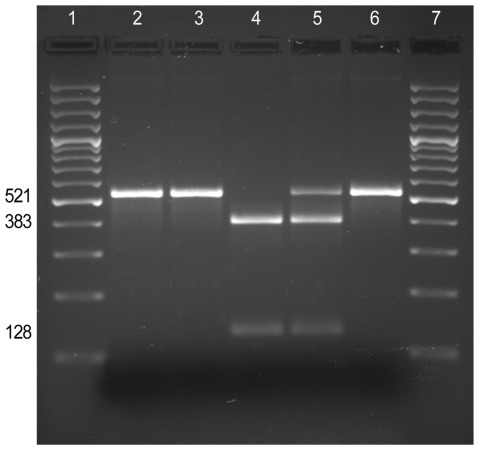
Screening for the A2047G mutation by PCR–restriction fragment length polymorphism analysis. The 521-bp fragment of the 23S rDNA gene amplified by PCR from the *Bordetalla pertussis* clinical isolates (FR4229, FR4930, and FR4991) and controls (A228 and Tohama I) was digested with the endonuclease BbsI. Lanes 1 and 7, M, 100-bp ladder (SM0321; Fermentas, St. Leon-Rot, Germany); lane 2, *B. pertussis* FR4929; lane 3, *B. pertussis* FR4930; lane 4, *B. pertussis* FR4991; lane 5, control *B. pertussis* A228 (erythromycin resistant, heterozygous); lane 6, control *B. pertussis* Tohama (erythromycin susceptible).

## Conclusions

The patient was brought for treatment with severe whooping cough which required ICU management and 2 courses of blood exchange. Nevertheless, *B. pertussis* resistance to macrolides is unlikely to be the cause of this severity because the infecting strain was found twice to be sensitive to all macrolides, and acquired resistance subsequently. However, despite appropriate treatments, we could not eradicate *B. pertussis*, and cultures were positive after 2 courses of macrolides and 1 course of cotrimoxazole given for an associated staphylococcal infection. Presumably, this prolonged carriage favored the acquisition of resistance.

This observation confirms the need for maintaining the ability to culture *B. pertussis* isolates to analyze the evolution of their characteristics as well as their antimicrobial drug resistance. Monitoring for macrolide resistance is thus essential when investigating individual treatment failures, in particular, in infants <6 months of age. The resistance mechanism we report is similar to that described for an isolate collected in the United States, but these findings do not rule out the potential for the emergence of alternative resistance mechanisms.
